# Refining HIV Risk: The Modifying Effects of Youth, Gender and Education among People Who Inject Drugs in Poland

**DOI:** 10.1371/journal.pone.0068018

**Published:** 2013-07-23

**Authors:** Michal Czerwinski, Louise-Anne McNutt, Jack A. DeHovitz, Andrzej Zielinski, Magdalena Rosinska

**Affiliations:** 1 Department of Epidemiology, National Institute of Public Health – National Institute of Hygiene, Warsaw, Poland; 2 Department of Epidemiology & Biostatistics, SUNY School of Public Health, Albany, New York, United States of America; 3 Department of Medicine, SUNY-Downstate Medical Center, Brooklyn, New York, United States of America; Johns Hopkins School of Public Health, United States of America

## Abstract

**Objective:**

The goal of this study was to examine specific factors placing young (aged <30) women who inject drugs at higher risk for HIV, and to establish the need for targeted interventions within this population.

**Methods:**

A national cross-sectional sero-survey was conducted in 2004–2005 in six regions in Poland. A snowball sample of ever-injectors was recruited from drug treatment facilities and the surrounding community. Log-binomial regression was used to estimate adjusted prevalence ratios (PRs).

**Results:**

A total of 491 injection drug users younger than 30 were recruited, of whom 159 were women and 332 were men. The prevalence of HIV was 16.4% and 9.6% among women and men, respectively. In multivariate analysis, young female injectors whose education terminated at the primary level were more likely to be HIV-positive compared to males with a similar level of education (PR = 3.34, 95% CI = 1.86–6.00) and more highly educated women (PR = 4.16, 95% CI = 2.21–7.82).

**Conclusions:**

This study confirms an elevated risk of HIV among under-educated young women. Suggestions for specific interventions to reduce HIV transmission are presented. Additional research is needed to quantify the differential distribution of risk behaviors which amplify their likelihood of transmission.

## Introduction

The Polish HIV/AIDS surveillance system was implemented in the mid-1980s as a component of the national infectious disease surveillance system. Today, the surveillance system tracks both newly detected HIV cases and incident AIDS cases according to the European case definition [1], [2]. The first observed HIV outbreaks among people who inject drugs (PWID) in Poland occurred in 1989, and since then injecting drug use (IDU) has played a pivotal role in fueling the HIV epidemic. In 1994, the published prevalence estimates of HIV among PWID were as high as 46% [3]. Despite a steady downward trend of IDU-acquired infections since 2003, a substantial proportion of HIV/AIDS cases remain in this population [4], [5], [6], [7]. By the end of 2010, PWID accounted for approximately 41% of the cumulative number of all HIV infections and 50% of reported AIDS cases [4]. While three in four HIV-positive PWID are male, the gender distribution is markedly different among younger (<25 years) HIV-positive PWID, among whom 47% are female. This differential gender distribution when stratified by age suggests that among PWID young female injectors are a particularly vulnerable group.

Recent studies have begun to describe this occurrence, indicating that among injectors, women (particularly adolescents) have a higher HIV incidence [8], [9] and, compared to males, females are more likely engage in needle borrowing, ancillary equipment sharing or being injected by someone else [10], [11], [12]. Female injectors are also more likely to have steady sex partners, with whom they also inject [12], [13]. Increased injection-related risk among females might be associated with initiation into injection drug use through male sex partners [14], and there appears to be substantial overlap between sexual and injection partnerships. Some studies indicate that the greater risk for HIV infection among female injectors might be associated with high-risk sexual behaviors (e.g., from unprotected sex) [11], [15] and engaging in sexual activity in exchange for drugs or money [16], [17], [18]. To examine factors related to this increased vulnerability of young women who inject drugs, we compared HIV prevalence between young male and female injectors to quantify the excess risk of HIV accrued by young female injectors, and ascertain whether this increased risk can be attributed to characteristics potentially amenable to intervention.

## Methods

### Ethics Statement

We analyzed data from a national, cross-sectional sero-survey conducted throughout six regions in Poland during 2004–2005. Respondents included ever-injectors recruited from drug treatment facilities and the surrounding community. This study was approved by the Institutional Review Board (IRB, Office of Regulatory Research Compliance) of the University at Albany, State University of New York.

The original survey and the informed consent process were approved by the IRB of the National Institute of Public Health – National Institute of Hygiene (NIPH-NIH), Warsaw, Poland. Potential study participants were provided information on the purpose of the study and their rights as a study participant, including the voluntary nature of participation and anonymity.

The survey was intended for ever-injectors aged 18 or older, but 19 participants were likely younger than 18 years based on self-reported birth date. These responses were retained for analysis upon approval by the IRB. All research participants indicated in writing their willingness and eligibility to participate by signing the consent form. To ensure anonymous participation, the study protocol did not involve authentication of survey respondents.

### Sample

The survey was designed for second-generation surveillance among PWID and targeted regions in Poland with the highest demand for dependence treatment or in the vicinity of highly affected areas (Kaliningrad and Ukraine). Participants were recruited from 15 locations in six out of 16 regions of Poland (Mazowieckie, Lubuskie, Slaskie, Dolnoslaskie, Lubelskie and Warminsko-Mazurskie).

Two methods were utilized to recruit participants to the study: (1) sampling at locations known to be frequented by PWID (e.g., parks, streets) and community-based programs serving PWID (e.g., needle exchange, methadone programs) combined with chain-referral (snowball sampling), and (2) recruitment at treatment. Using a classic chain-referral method, we asked each participant to refer acquaintances who met the study eligibility criteria. Referrals were also requested from subsequent participants. In regions which did not enroll the desired sample through chain-referral, participants were also recruited at treatment facilities (e.g., detoxification wards and other in-patient treatment centers) where all eligible PWID were invited to participate in the study.

The following criteria was used to select participants: (1) injection drug use of at least one illicit drug in their lifetime, (2) residence for at least 3 months prior to the study interview in the geographic region, (3) willingness to complete a survey, and (4) consent to testing for HIV, hepatitis B virus (HBV) and hepatitis C virus (HCV). To investigate recent risk behaviors among individuals recruited in treatment facilities, treatment must have been for less than three months.

### Study Procedures

We provided potential study participants with information regarding the objectives of the study and their rights as a study participant, including the voluntary nature of participation and anonymity. All participants provided a blood sample for HIV.

*The questionnaire* was designed in the Department of Epidemiology NIPH-NIH, using existing behavioral surveillance indicators. It was written in plain language in consultation with harm reduction personnel and pilot-tested among the target group. Although the questionnaire was originally designed for a self-administered survey, respondents were encouraged to meet face-to-face with trained interviewers to ensure accuracy and completeness of the data. The questionnaire contained closed-ended questions on socio-demographic characteristics, injection behaviors, sexual behaviors and other risk factors.

*Socio-demographic characteristics* included: gender, age, characteristic of family (cohabiting with partner, spouse, or parents) and living conditions, education, employment, source of income, history of homelessness, imprisonment or arrest.

*Injecting risk characteristics* were solicited, including: (1) history of injecting drug use (e.g., age of illicit drug initiation, duration of injection, drug(s) of choice, frequency of drug administration (especially illicit drugs on a daily basis), and route of drug administration); (2) injecting practices (e.g., history of sharing needles, syringes, filters, usage of clean syringes and needles, method of disinfection when re-using needles, history of syringes, needle and filter sharing with known HIV- and HCV-positive persons); (3) participation in needle/syringe programs; and (4) history of drug treatment (e.g., counseling in addiction treatment center, drug rehabilitation and detoxification).

*Sexual risk characteristics* included questions on the number of sexual partners in the last 12 months, sex with a person of the same gender, sex with an IDU, payment for sex, exchange of sex for drugs or money, and consistent usage of condoms.

### Laboratory Methods

Serum samples taken from all participants were screened for HIV antibodies using commercial enzyme immunoassay kits (Abbott and Organon Teknika). We followed WHO recommendations for HIV surveillance testing strategies [19], and all specimens were tested twice. The highly sensitive and specific tests selected for screening were combined HIV-1/HIV-2 assays.

### Statistical analysis

The analysis was performed on a subset of the original dataset: respondents younger than 30 years of age.

#### Univariate analysis

The association of gender with the following characteristics was evaluated with Chi-square tests of the difference in proportions: (1) seroprevalence of HIV, (2) basic sociodemographic factors, and (3) injecting and sexual risk behaviors. For expected cell sizes of five or fewer, Fisher's exact test was employed. To examine differences for continuous variables, we used the Wilcoxon rank-sum test. For some analyses, stratification was based on age, level of education, characteristics of the family, history of imprisonment, knowledge of HIV status or recruitment method.

#### Multivariate analysis

Log-binomial regression was used to model HIV prevalence by risk behaviors [20]. Analyses focused on estimating the association between gender and HIV prevalence, after adjustment for confounding by known risk factors. Effect modification (interaction with gender) was evaluated for recognized risk factors for HIV infection. Therefore, in addition to gender, the initial model included time since IDU initiation (IDU duration), needle sharing and sex with a drug-using partner. Any variable which interacted significantly with gender (*p*<0.05) was considered an effect modifier.

Variables were entered into the model in a stepwise fashion using results of the univariate analysis (*p*<0.2). The following covariates were considered for model entry: age, education (primary school, vocational school, secondary education, higher education), employment (full or part time job, student, jobless, other), characteristic of family (cohabiting with a steady partner or spouse, parents, relatives, children, friends, PWID, living alone), homelessness ever (currently, within last 12 months, over one year ago, never), imprisonment ever (yes, no), knowledge of HIV status (yes, no), opiates (yes, no), age at illicit drug initiation (<19 years, ≥19 years), duration of injection (less than 2 years ago, 2–5 years, 6–10 years, more than 10 years ago), daily injection ever (yes, no), receptive sharing including needles, syringes or filters (within last 30 days, more than 30 days ago to 2 years ago, more than 2 years ago, never), number of sexual partners in last 12 months (no partner, 1 partner, multiple partners), sex with a drug-using partner ever (yes, no), payment for sex (in last 12 months, not in last 12 months, never), sex for drugs and consistent usage of condoms (yes, no).

To improve the convergence of the tested models, independent variables with more than two categories were usually dichotomized, and the COPY method described by Deddens and Petersen [21] was utilized. It is important to note, however, that the final model converged on the original data set, and all presented prevalence ratios were estimated on the underlying data. Analysis was performed using SAS 9.2.

## Results

### Socio-demographic characteristics

Our study population comprised 491 PWID younger than age 30 of whom 159 were women and 332 were men. Overall, women tended to be recruited on streets and at community-based programs, however there were noticeable differences between the regions (Table S1). Compared to males, women were better educated, and more likely to cohabit with another person who injects drugs. However, they were less likely to have a prison history or to be currently undergoing addiction treatment. (Table 1)

**Table 1:** Sociodemographic characteristics of young (<30) female compared to young male PWID in a cross-sectional study conducted in Poland, 2004–2005.

Characteristics		Females		Males		*P* value
		n = 159	(%)	n = 332	(%)	
Enrollment*	in-patient	39	(24,5)	132	(39,9)	0,001
	out-patient	120	(75,5)	199	(60,1)	
Age	16–19	19	(12,0)	31	(9,3)	0,388
	20–24	81	(50,9)	158	(47,6)	
	25–29	59	(37,1)	143	(43,1)	
Education	primary school	46	(28,9)	127	(38,3)	<0,001
	vocational school	30	(18,9)	100	(30,1)	
	secondary education	60	(37,7)	88	(26,5)	
	higher education	23	(14,5)	17	(5,1)	
Employment	full, part time job	25	(15,7)	77	(23,3)	0,009
	student	33	(20,8)	35	(10,6)	
	jobless	85	(53,5)	175	(52,8)	
	other (incl. retirement)	16	(10,0)	44	(13,3)	
Living with**	spouse/partner	35	(22,0)	43	(13,0)	0,011
	parents/relatives	81	(50,9)	176	(53,2)	0,644
	children	19	(12,0)	6	(1,8)	<0,001
	friends	25	(15,7)	31	(9,3)	0,037
	PWID	112	(70,9)	183	(55,1)	0,001
	on my own	10	(6,3)	26	(7,9)	0,534
Homeless ever	yes, currently	12	(7,6)	24	(7,2)	0,691
	within last 12 months	15	(9,4)	29	(8,7)	
	over one year ago	24	(15,1)	65	(19,6)	
	no, never	108	(67,9)	214	(64,5)	
Imprisoned ever		38	(23,9)	158	(47,6)	<0,001

* in-patient = in-patient treatment facilities and programs; out-patient = community-based programs serving PWID and surrounding community.

** may be in more than one category.

### Prevalence of risk behaviors

In the *univariate analysis*, young (<30 years) females and males were similar in terms of injection risk behavior (Table 2) but differed in two areas with respect to their sex risk behaviors. (Table 3) While both females and males reported a median of 1 (IQR = 1, 3) recent sex partners, female injectors more often reported sexual intercourse with a drug-using partner (70.4% of females vs. 48.4% of males, *p*<0.001), and a higher proportion of females reported engaging in sex in exchange for money or drugs. (Table 3)

**Table 2:** Injection risk behaviors of young (<30) female compared to young male PWID.

Risk behaviors		Females		Males		*P* value
		n = 159	(%)	n = 332	(%)	
Initiation of injecting, years	less than 2 years ago	43	(27,9)	66	(21,6)	0,414
	2–5	65	(42,2)	131	(43,1)	
	6–10	36	(23,4)	88	(29,0)	
	>10	10	(6,5)	19	(6,3)	
Age began injecting, median (IQR)		18	(16, 21)	19	(17, 21)	0,513
Recent injection	last 30 days	76	(49,4)	132	(42,3)	0,355
	>30 days - 5 years	74	(48,1)	171	(54,8)	
	>5 years ago	4	(2,5)	9	(2,9)	
Daily injection ever	yes	124	(79,5)	271	(83,4)	0,296
	no	32	(20,5)	54	(16,6)	
Shared/borrowed needles/syringes	within last 30 days	15	(9,8)	34	(10,6)	0,206
	>30 days - 2 years	39	(25,5)	100	(31,3)	
	>2 years ago	27	(17,6)	68	(21,3)	
	no, never	72	(47,1)	118	(36,8)	
Usage of any disinfectant	never	111	(83,5)	226	(79,0)	0,558
(e.g. chlorine)*	less than in half of injections	10	(7,5)	20	(7,0)	
	at least in half of injections	4	(3,0)	14	(4,9)	
	always	8	(6,0)	26	(9,1)	
Shared ancillary equipment**	never	58	(46,8)	105	(39,5)	0,409
	less than half of injections	45	(36,3)	98	(36,8)	
	at least half of injections	14	(11,3)	43	(16,2)	
	always	7	(5,6)	20	(7,5)	

* when injecting with borrowed needles/syringes.

** when injecting with new needles/syringes.

**Table 3:** Sexual risk behaviors of young (<30) female compared to young male PWID.

Risk behaviors		Females		Males		*P* value
		n = 159	(%)	n = 332	(%)	
No. of sex partners (in last 12 month), median		1	(1, 3)*	1	(1, 3)*	0,268
No. of sex partners (in last 12 month)	0	17	(11)	68	(20,5)	0,027
	1	64	(41,3)	112	(33,8)	
	2 and more	74	(47,7)	151	(45,7)	
Consistent usage of condoms (lifetime)	no	121	(76,1)	277	(83,4)	0,052
	yes	38	(23,9)	55	(16,6)	
Engaged in sex for drugs	in last 12 months	18	(11,3)	6	(1,8)	<0,001
	not in last 12 months	15	(9,4)	8	(2,4)	
	never	126	(79,3)	318	(95,8)	
Engaged in sex for money	in last 12 months	25	(15,7)	10	(3,0)	<0,001
	not in last 12 months	20	(12,6)	16	(4,8)	
	never	114	(71,7)	306	(92,2)	
Had an IDU sex partner	yes	112	(70,4)	161	(48,4)	<0,001
	no	47	(29,6)	172	(51,6)	

* interquartile range.

### Prevalence of HIV infection and predictors

**Figure 1:**
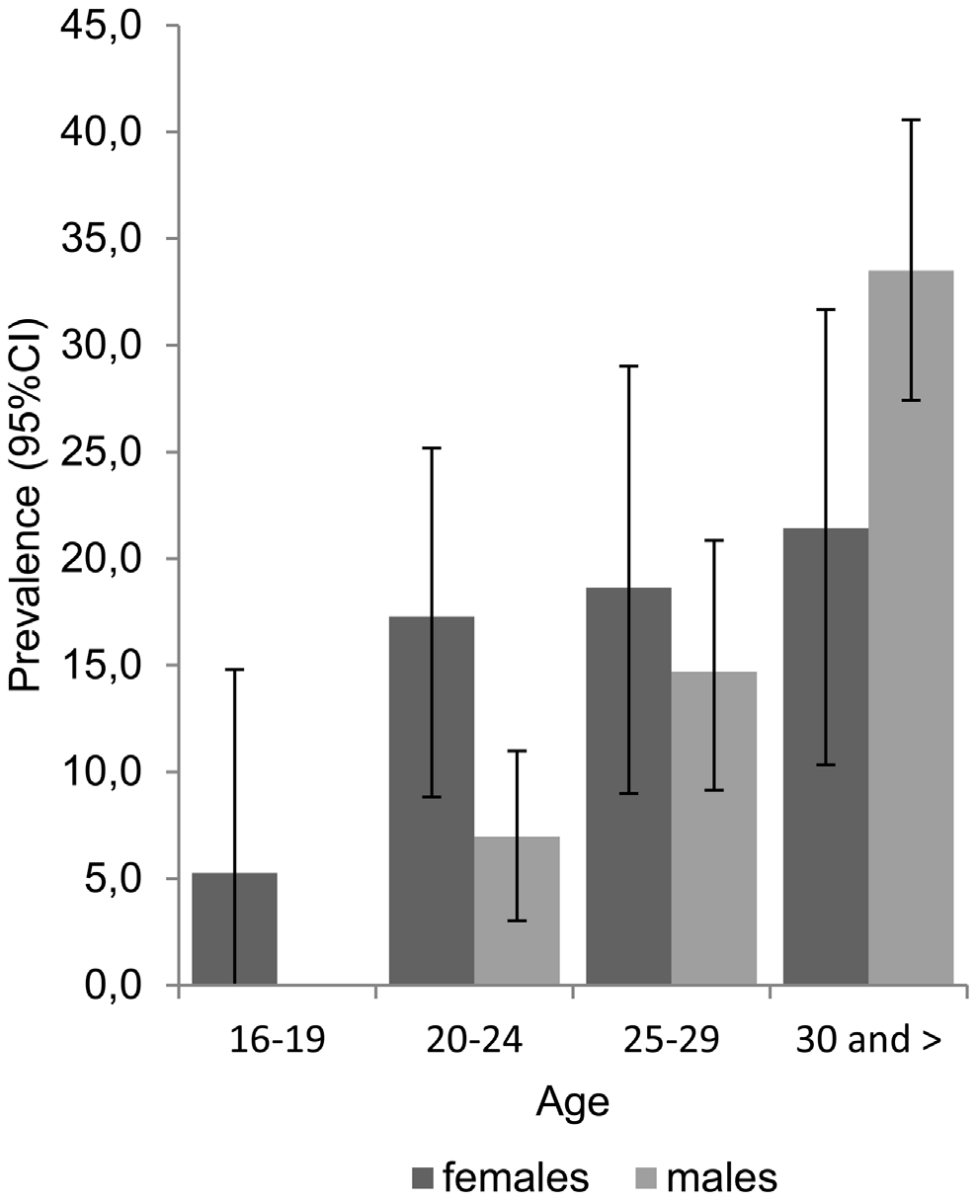
HIV prevalence with 95% confidence intervals stratified by gender and age, in a cross-sectional study conducted in Poland, 2004–2005. Of the 748 respondents to the original survey, 137 (18.3%) had a positive test result for HIV. Upon age stratification, females in younger age groups tended to have a higher level of HIV infection compared to males. Overall, HIV prevalence among females and males aged <30 years (analyzed subset of the original dataset) was 16.4% and 9.6%, respectively. Among those ≥30 years, the relative proportions were reversed: HIV prevalence was 21.4% among females and 33.5% among males.

HIV prevalence among young (<30 years) women and men was 16.4% and 9.6%, respectively. Upon age stratification, females in younger age groups tended to have a higher level of HIV infection compared to males. (Figure 1) When stratified by low level of education (only primary education – yes vs. no), females (<30 years) with primary education were almost three times (PR = 2.96, 95% CI = 1.55, 5.64) more likely to be HIV-positive compared to a similar group of males; HIV prevalence among this group of women was nearly 33%.

In multivariate analysis (the log-binomial regression used 440 observations), gender, education, and duration of IDU were significantly associated with HIV infection; cohabiting with spouse and sex with drug-using partner approached statistical significance. Low level of education (highest grade of completion = primary school, 8 years of education) was a significant predictor for HIV. The log-binomial model also indicated an interaction between gender and education. The magnitude of effect for low education was clearly different between males and females. Women with an education limited to the primary school were much more likely to be HIV-positive compared to males with a similar education (PR = 3.34, 95% CI = 1.86, 6.00) and more highly educated women (PR = 4.16, 95% CI = 2.21, 7.82). However, young female and male injectors cohabiting with a steady partner had a lower risk for HIV infection for all education strata. (Table 4)

**Table 4:** Unadjusted prevalence ratios (PR) calculated in univariate analyses and adjusted prevalence ratios (APR) estimated in log-binomial model fitted for young (<30) PWID, with 95% confidence intervals (CI).

Stratum	Factor		Univariate analyses				Multivariate analysis*	
			n	n HIV+	(%)	PR (95% CI)	APR (95% CI)	*P* value
Primary	Gender	males (ref.)	127	14	(11,02)	1	1	<0,001
sch. only		females	46	15	(32,61)	2,96 (1,55; 5,64)	3,34 (1,86; 6,00)	
> primary	Gender	males (ref.)	205	18	(8,78)	1	1	0,600
sch.		females	113	11	(9,73)	1,11 (0,54; 2,26)	1,21 (0,59; 2,50)	
Females	Education	> primary sch. (ref.)	113	11	(9,73)	1	1	<0,001
		primary sch. only	46	15	(32,61)	3,35 (1,67; 6,74)	4,16 (2,21; 7,82)	
Males	Education	> primary sch. (ref.)	205	18	(8,78)	1	1	0,223
		primary sch. only	127	14	(11,02)	1,26 (0,65; 2,44)	1,51 (0,78; 2,93)	
Total	IDU	<5 years ago (ref.)	109	2	(1,83)	1	1	0,001
	initiation	≥5 years ago	348	55	(15,80)	8.61 (2,14; 34,73)	9,77 (2,45; 39,00)	
Total	Needles	never (ref.)	190	20	(10,53)	1	1	0,390
	sharing	ever	282	38	(13,48)	1,28 (0,77; 2,13)	1,23 (0,77; 1,95)	
Total	Sex with	never (ref.)	218	13	(5,96)	1	1	0,101
	PWID	ever	273	45	(16,48)	2,76 (1,53; 4,99)	1,65 (0,91; 3,00)	
Total	Steady	no (ref.)	412	51	(12,38)	1	1	0,067
	partner	yes	78	6	(7,69)	0,62 (0,28; 1,40)	0,49 (0,23; 1,05)	

* The final log-binomial model used to estimate APRs included the following terms: Gender (females vs. males), Education (primary vs. more than primary sch.), IDU initiation (<5 years ago vs. ≥5 years ago), Needle sharing (ever vs. never), Sex with PWID (ever vs. never), Cohabiting with spouse/partner (yes vs. no) and an interaction term between Gender and Education. Due to significant interaction between Gender and Education, the effect of Gender is presented separately for lower (primary school) and better educated (more than primary school) study participants, just as effect of Education is presented separately for females and males.

## Discussion

In this study of young Polish PWID, young female injectors had a higher prevalence of HIV infection compared to males. Limited education magnified the effect of gender on HIV risk. This finding is consistent with previous studies which have suggested a higher risk for blood borne viral infections among young females, who inject drugs [22], [23], [24]. The relationship between educational level and HIV is less clear. Specifically, several studies have reported an association between lower educational attainment and increased risk of HIV [15], [25], [26], [27], [28], but others have found no effect [13]. Our study shows that education has a differential impact on HIV risk by subgroup of PWID, and we quantify the role of limited education in amplifying the disproportionate risk borne by young female injectors.

When compared to male peers, the level of education among female injectors was a strong predictor of HIV infection, even after adjusting for injection and sex risk behaviors. Women from this group were much more likely to be HIV-positive compared to both males with a similarly low level of education (APR 3.34) as well as more highly educated women (APR 4.16). However, we did not identify any particular risk behaviors in this group of young, undereducated, injecting women which could help explain their excess risk for HIV infection. Males and females had a comparable prevalence of injection risk behaviors, and the level of risk for HIV infection associated with those behaviors was similar among men and women. Over 70% of young female injectors reported sex with a drug-using partner compared to 48% of male PWID. This factor was not significantly associated with increased HIV risk in multivariate analysis, but it is possible that differential risk of HIV among female injectors could be explained by substantial overlap between sexual and injection partnerships and equipment sharing between partners [13]. It is possible that the lower educational attainment among women who inject drugs parallels their social context for drug use. Young, uneducated and unskilled women, may be forced to rely on male partners for support and accessing the drugs, what decreases their capability of negotiating safer injection and sex behaviors [29]. Additionally, they may be less exposed to prevention messages, be it at school or through accessing internet campaigns.

Yet despite the clear need for wrap-around services to address the complex needs of these young women, there is insufficient coverage of harm reduction services for PWID in Poland. In 2008, 11 harm reduction programs served a total of 3101 clients (of whom 22% were women) of an estimated 100 000 or more problem drug users in Poland [6], [30]. The coverage of opioid substitution therapy (OST) also remains low. In 2009, only 7% of problem opioid drug users received this form of treatment [31]. In Poland, PWID may also not benefit from relevant health programs including antiretroviral treatment (ART). Despite universal access to ART in Poland, relative excess of new AIDS diagnoses among persons infected through drug use may point to difficulties in achieving good ART coverage in this group [4].

In addition, structural barriers, such a strict anti-drug regulations, and a disconnect between services offered and the needs of the target population may exist, which discourage people from accessing harm reduction services. This may be particularly relevant for young PWID, as a study among clients of existing in Poland harm reduction programs have shown that 82% of them is aged 25 or above [30]. Previous studies concur that recruitment of young PWID through established outreach is complicated, and their needs are not met by traditional interventions [32]. Effective HIV prevention in this group requires a more integrated approach, such as establishing a matrix of comprehensive services which respond to specific needs, including gender specific needs, while reducing the social and economic barriers which hinder access [33], [34].

In Poland, community-based programs serving PWID focus on needle exchanges, but often do not offer more complex or comprehensive care. Very few harm reduction programs offer HIV, HCV or other sexually transmitted infection diagnosis or treatment. The services targeting female PWID are even more limited. Only four city programs serve female injecting sex workers, and none of the sites provide care specific to the health needs of women, such as reproductive care [30]. Expansion of these additional services may prove helpful in reaching young females who inject drugs and may not be concerned with harm reduction as such. An additional value of such approach would be to reduce the stigma of seeking care at a program solely focused on, for instance, needle exchange.

Successful harm reduction and health promotion programs often include peer outreach interventions to reach the PWID, who are not motivated enough to seek health services [35]. This may be due to social hardship leading to prioritization of basic needs and acquisition of drugs over the long-term risks. Social isolation might be a particular challenge among the young women identified here with low educational attainment. Peer counseling could provide these women and their partners with a better understanding of the unique risks associated with the overlap between sexual and injection partnerships. Finally, referrals should be provided to service organizations for nutrition support and government-sponsored agencies for behavioral health and vocational training [35].

### Limitations

Our study relied on convenience sampling design, making it difficult to assess potential selection bias. In order to reach different subgroup of PWID recruitment in different settings was introduced. However, the recruitment setting could confound the associations between gender, HIV prevalence and it's predictors. In our study, women tended to be recruited at community-based programs serving PWID and the surrounding community while men were more frequently recruited from in-patient drug treatment centers. However, the prevalence of injection and sexual risk behaviors were comparable for women across recruitment settings. Most importantly, lower educational attainment was consistently associated with an increased risk for HIV. We also note that by including the in-patient treatment centers, we were able to reach a less marginalized - in terms of employment and/or stable housing - group among the PWID.

Secondly, the data were collected with differing methods (self-administered vs. interview), but similar options were provided to all respondents in order to increase the accuracy of their responses and trained interviewers were on hand to facilitate comprehension of the survey.

Next, we analyzed baseline data, collected for surveillance purposes rather than to examine subtle differences in behavioral risks. As a result, some behavioral variables, which could have provided us better insight into the nature of excess risk for HIV infection among young female injectors were not available, including injection risks (e.g., being injected by someone else, injecting drugs in the context of a sexual partnership, having much older drug-using sex partners) or demographic characteristics (such as transient worker status) [10], [12], [36]. Additionally, stratification produced very small sample sizes which reduced our power to ascertain the individual contributions of available covariates to the risk relationship.

Finally, we recognize that the seven-year lapse since data collection is a drawback. However, the current distribution of age and gender for new HIV diagnoses among injecting drug users reported to the national surveillance registry has remained stable, suggesting a persistent excess risk for HIV among young women. In the most recent 5 years (2008–2012), 47,4% of reported HIV cases among young (<25 years) PWID are women (unpublished data, NIPH-NIH). Moreover, no specific prevention programs have been implemented targeting young women who inject drugs suggesting that the situation is unlikely to have changed substantially in this group.

## Conclusions

This study suggests that young undereducated women who inject drugs in Poland are at increased risk of HIV and would benefit from specific interventions to reduce HIV transmission. Intervention strategies must couple what we know about risk factors with the relevant and motivating social factors which compel these women to place themselves at risk for HIV. Focused research among this demographic may yield insight into additional factors which amplify the risk for HIV among young female injectors and help refine interventions aimed at this group.

## Supporting Information

Table S1 Recruitment setting of young (<30) female compared to young male PWID in a cross-sectional study conducted in Poland, 2004–2005.
(PDF)
