# Supplementary material for: Refining HIV Risk: The Modifying Effects of Youth, Gender and Education among People Who Inject Drugs in Poland
Source: PLoS One. 2013 Jul 23;8(7):e68018. doi: 10.1371/journal.pone.0068018 (PMC3720710; doi:10.1371/journal.pone.0068018)
Supplement: Table S1 — Recruitment setting of young (<30) female compared to young male PWID in a cross-sectional study conducted in Poland, 2004–2005. (PDF) [file pone.0068018.s001.pdf]

Table S1. Recruitment setting of young (<30) female compared to young male PWID in a cross-sectional study conducted in Poland, 2004 -2005.

Region	Recruitment setting*	Females		Males		*P* value
		n=159	(%)	n=331	(%)	
Lubelskie	in-patient	2	(12,5)	8	(44,4)	0,063
	out-patient	14	(87,5)	10	(55,6)	
Warminsko-Mazurskie	in-patient	0		42	(72,4)	0,009
	out-patient	4	(100)	16	(27,6)	
Lubuskie	in-patient	27	(73,0)	72	(92,3)	0,005
	out-patient	10	(27,0)	6	(7,7)	
Slaskie	in-patient	0		0		
	out-patient	18	(100)	19	(100)	
Mazowieckie	in-patient	0		0		
	out-patient	43	(100)	106	(100)	
Dolnoslaskie	in-patient	10	(24,4)	10	(19,2)	0,547
	out-patient	31	(75,6)	42	(80,8)	

* in-patient = in-patient treatment facilities and programs
out-patient = community-based programs serving PWID and surrounding community
